# Epidemic Diseases

**Published:** 1866-11

**Authors:** F. B. Haller

**Affiliations:** Vandalia, Ill.


ARTICLE XL.

### EPIDEMIC DISEASES.

By F. B. HALLER, M.D., Vandalia, Ill.

Read to the Illinois State Medical Society, June, 1866.

Upon my return home, about the 1st of April, 1865, I found an epidemic of erysipelas, rubeola, and pertussis, all of which were of a rather mild type, when uncomplicated; but, unfortunately, many of the measle cases were complicated with pneumonia, which rendered them exceedingly difficult to manage. One case that I saw in consultation, proved fatal. In a majority of the cases, the supporting and stimulating treatment had to be resorted to at the very onset of the attack, and in every instance was it required at some particular stage of its progress. Those of an uncomplicated nature, that I was consulted about, were left to good nursing and hygienic means, and made excellent recoveries, much better than those that were unfortunately drenched by their good mothers with the various panacea teas, the most popular of which is the celebrated sheep-tea, which the little fellows had to drink in large quantities, to the shame of their mothers.

There was nothing unusual in the cases of whooping-cough that occurred. In this disease, I used the bromide of ammonia with apparently good effect, and, from my experience with it, am led to believe it an excellent remedy in this disease, when uncomplicated.

The erysipelatous cases presented nothing of interest. They all readily yielded to ordinary treatment with iron and quinia. During the months of April, May, and June, there were a great many cases. About this time, I had several cases requiring surgical operations, and in every single instance, notwithstanding I used every precaution to prevent it, I had traumatic erysipelas to contend with, and came very near losing two of my patients.

Since June, there has been only an occasional case. As these epidemics began to subside, bilious fevers set in, and by the middle of July, we were into one of the most prevalent epidemics of malarial diseases that has visited this vicinity during the last sixteen years, the time of my sojourn here, and, as usual when we have a good deal of malarious fevers, they were a mild type, and readily yielded to the proper remedies. Ever since then, throughout the entire winter and spring months, have we been having a great many cases of remittent, intermittent, and pernicious fevers, of a much graver character than occurred during the months of summer and autumn. There was this striking peculiarity about these fevers throughout this epidemic, that they required at least one-third larger doses to subdue them than they had at any former period, since I have been practicing in this locality, not only of antiperiodics, but of purgatives, opiates, or any other medicines that might be indicated in particular cases.

Never, during my residence of sixteen years in this place, have I seen these fevers so prevalent in winter and spring months, and the oldest citizens inform me that they never saw the like before. During this epidemic, I have used more mercurials than I ever did before, nor can I call to mind a single instance where a patient was ptyalized, beyond a mere touch of the gums. I am firmly of the opinion that by the free use of this potent remedy I was enabled to relieve my patients much sooner and had them more thoroughly cured, than by any other means I had at my command. Indeed, so happy were the effects of this remedy upon my patients, that for a time I had almost become an old fogy routinest.

During the last summer, we had but very little summer complaint amongst the children, less than ever I saw before, and what few cases there were were easily managed. I do not think we lost a single patient with this disease during the whole season.

During the autumn months, variola made its appearance in a neighborhood about 15 miles south-east of our place, which soon became epidemic, and by the beginning of winter, many of the families in that region had it. Quite a number of these cases were of a malignant character, and only yielded to the most vigorous treatment. A goodly number of these cases were under the care of my partner, Dr. R. S. HIGGINS, from whom I obtained this information.

In January, it broke out simultaneously in our village and several adjoining neighborhoods, and continued for some time, and, as it was in the first neighborhood, quite a number of the cases were of a malignant nature. In a shanty in the west part of the town, which was poorly ventilated, nearly half those attacked died; and at the poorhouse, amongst the paupers, there were several deaths. What treatment or care these persons received I have no means of knowing.

The other cases were treated, mostly, by my partner, Dr. R. S. HIGGINS, who informs me that when desquamation began, and in some instances even before this stage, he had to resort to the supporting and stimulating treatment, by which method he was exceedingly fortunate, not losing a single case out of the many he treated. The few cases that fell into my hands were of a mild type, and required no treatment but an occasional purgative of castor-oil, and good nursing.

During this epidemic, we have had the very best of evidence in proof of the prophylactic effects of vaccination. There were no sanitary regulations, nor any means used to prevent its spread; nurses, and those who had varioloid in a mild form, mingled promiscuously with the people on the streets and in the avenues of business, and the only thing that was done to guard against its spread was vaccinating and re-vaccinating, which was pretty thoroughly done. Notwithstanding this free mingling of nurses, and those slightly affected, with the populace, it was not communicated in a single instance; and only in a few instances, did those who were exposed as nurses, or otherwise, who had been previously vaccinated, take varioloid. In one family of twelve, all living in one room, is proof most emphatic as to its prophylactic powers; here, an infant died, and the father had it in a malignant form, and not one of those who had been previously vaccinated had it in any form—were perfectly exempt from it, notwithstanding they were constantly in this one room, cooked, eaten, and slept there. There were several instances nearly as striking as this. Certainly, these are strong proofs in favor of vaccination and re-vaccination, and it seems to me, all that is necessary to entirely free the human race from this scourge, is to compel every person to be vaccinated and re-vaccinated until it will take no more, and then inoculate with the pure variola virus. By this means, the system would become so thoroughly protected against it, that it would be utterly impossible for them to ever have it, even in the most mild form.

We have had much less pneumonia this winter and spring than usual, but the few cases that have occurred were more difficult to manage than common, many of them resulting fatally. Nearly all the cases that fell under my observation were asthenic in character, requiring beef-tea and stimulants from the beginning of the attack pretty freely. There was, in most of these cases, a marked tendency to congestion of the viscera of the abdomen, and, in a few instances, to spinal meningitis. These cases usually required large doses of quinine, until they were thoroughly under its influence.

Amongst the children that were attacked, during the months of February and March, with lung diseases, there was spinal meningitis in nearly or quite every case that came under my observation. These cases I treated with calomel, tartar emetic, and ipecac until the inflammatory action had subsided, when I put them upon full doses of chlorate of potassa and sulph. of quinine. During the whole period of this disease, up to the establishment of convalescence, I had rubefacients or blisters applied to the spine, with the most salutary effect. All my patients recovered, except one who was poorly cared for.

The past year has been one of great extremes of weather, such as was well calculated to produce more than the ordinary number of cases of sickness. For the last few months we have been having a great deal of easterly winds, more than I recollect to have ever seen before, which I think has been the cause, to a great extent, of the obstinacy of our diseases. Our very old people and those of feeble constitutions have suffered more in portion than the young and vigorous, as compared with other springs.

We have had less typhoid fever this last year, than for many years past, although there were a great many cases of remittent fever that assumed a typhoid character, and such cases were usually protracted and had to be strongly supported. These cases I gave beef-tea and milk *ad libitum.* The result was very satisfactory. Nearly all our cases for the past year have been of an asthenic character. This is becoming more and more so every year.

The lancet is scarcely ever used. Digitalis, tartar emetic, or veratrum viride are only in a few instances called into requisition. Opium will control the inflammatory action in most cases, and with it my patients make more rapid recoveries than under the more active treatment.

This, sir, is about a true character of the diseases that have occurred in our locality during the past year, given in a hasty, desultory manner.

